# *Potentilla reptans* L. preconditioning regulates H19 and MIAT long noncoding RNAs in H9C2 myoblasts Ischemia/Reperfusion model

**DOI:** 10.1186/s12906-023-04071-z

**Published:** 2023-07-31

**Authors:** Hassan Mirzaei, Aref Salehi, Bita Javan, Ayesheh Enayati, Morteza Olad nabi, Mehdi Zahedi, Gokhan Zengin

**Affiliations:** 1 Ischemic Disorders Research Center, Golestan University of Medical Sciences, Gorgan, Iran; 2 Medical Cellular and Molecular Research Center, Golestan University of Medical Sciences, Gorgan, Iran; 3 Department of Biology, Science Faculty, Selcuk University, Konya, 42130 Turkey

**Keywords:** Myocardial Ischemia/Reperfusion, Hypoxic preconditioning, *P. reptans*, *lncRNA H19*, *lncRNA MIAT*, H9C2

## Abstract

The present study aimed to evaluate the effect of the ethyl acetate fraction of *P. reptans* root (PEF) preconditioning on expressions of lncRNAs H19 and MIAT in H9C2 myoblasts I/R injury.

H9C2 cells were treated with different concentrations ranging from (10–400 µg/ml) of PEF for 24 h, followed by simulation of I/R condition. For I/R experiments, H9C2 cells were subjected with the oxygen and glucose deprivation for 2 h.

H9C2 cell viability was significantly enhanced by PEF preconditioning under I/R condition in a concentration-dependent manner up to 200 µg/ml as a EC_50_. The PEF significantly diminished the expression of lncRNA MIAT and rate of apoptosis against the I/R group. In addition, PEF pretreated before stimulation I/R condition increased H19 expression compared to the normal PEF group with no statistically significant differences between groups. Hence, the results suggest that PEF can protect cardiomyocytes during hypoxia-induced myocardial cell injury by targeting specific involved genes.

## Introduction

Cardiovascular diseases, especially the ischemic heart disease, are the most important causes of death in the world. A growing body of evidence indicated that preconditioning phenomenon is an effective way to protect heart against I/R injuries [1, 2]. However, regarding the lack of prediction of sustained occlusion of a coronary artery this phenomenon limits to use in clinical practice [3]. Various studies have indicated cardioprotective effects of natural products and medicinal plants [2, 4]. Therefore, finding natural pharmacological agents with a reliable preconditioning activity could be valuable to prevent destructive I/R injuries.

*Potentilla reptans* L*.*, Rosaceae family, has been traditionally used in Asia, Europe, and North America [2]. Recent updates, the cardioprotective effects of *Potentilla* genus has been assessed for their in vitro and in vivo studies in hypoxic and ischemic conditions [2, 5–9]. Likewise, Enayati et al. showed the ischemic preconditioning and postconditioning effects of ethyl acetate fraction of *P. reptans* root via its antioxidant and anti-apoptotic properties through NO release, activation of Nrf2 and RISK/SAFE pathways, as well as inhibiting GSK-3β and SGK1 protein kinases [2, 4]. Although some cardioprotective effects of *P. reptans* have been indicated, it seems that due to I/R triggers other signaling cascades and gene/proteins, it is necessary to investigate the other related *P. reptans* cardioprotective signaling molecules.

Long ncRNAs (lncRNAs) are the most prevalent class of non-coding RNAs (ncRNAs) with more than 200 nucleotides. They closely play a key role as signaling molecules in mitochondrial influence of I/R injury [10, 11]. The lncRNA H19 (H19) and lncRNA myocardial infarction-associated transcript (MIAT) which are mainly expressed in the heart [12, 13]. More recently, H19 has shown a protective effect through increasing its expression against surgically or hypoxia-induced I/R injury in cardiomyocytes [8, 11]. Numerous studies indicated that H19 associated with hypertension, coronary artery disease, atherosclerosis, MI, and heart failure [14, 15]. However, H19-mediated regulation of cardiac dysfunctions in the different cardiac pathological processes through directly or indirectly different signaling pathways and molecular targets [15]. In addition, MIAT is another type of lncRNA, which is involved in I/R injury induced cardiomyocytes necrosis, apoptosis, cell proliferation, and fibrotic remodeling [10]. Likewise, increased cardiac MIAT expression following I/R exerts its pro-hypertrophic and the complex regulatory network effects via wiping the anti-hypertrophic miRNAs including miR-150, miR-93 and miR-93 in cardiomyocytes [12, 16–18]. Interestingly, knockdown of MIAT prior to myocardial ischemia preserved cardiac against I/R injury and fibrosis or apoptosis by reducing infarct size and improving cardiac function [10]. Therefore, the current study investigated the preconditioning and anti-ischemic effects of PEF in accordance with its underlying effects on the expression of H19 and MIAT in the H9C2 myoblasts I/R injury along with evaluating their molecular targets by Molecular Docking.

## Methods

### Plant collection and identification

*P. reptans* roots were collected from the North region of Iran. Plant specimens were deposited at the Central Herbarium, Tehran University, Iran, under the voucher specimen under the voucher number 45815-TUH [2] and plant specimens were identified by a botanist, Dr. Farideh Attar.

### Preparation of plant extract

The extraction and fractionation process of *P. reptans* root were described as previously [2]. The *P. reptans* ethyl acetate fraction was dissolved and sonicated in DMSO.

### Cell culture

The H9C2 cells, rat cardiomyocyte cell lines, were obtained from the Cell Bank of the Pasteur Institute, Tehran, Iran. Cells were maintained in high glucose Dulbecco's modified Eagle's medium (DMEM) supplemented with a 10% (v/v) fetal bovine serum (FBS) and 1% penicillin/streptomycin, in a CO_2_ incubator at 37 °C.

### Cell viability assay

The cell viability was detected using the colorimetric 3-[4,5-dimethylthylthiazol-2-yl]-2,5 diphenyl tetrazolium bromide (MTT) assay. Briefly, cells were seeded into 96-well plates at the density of 104 cells/well and incubated for 24 h and then were pre-treated with a range of PEF concentrations (10, 50, 100, 200, 300, 400 µg/ml) for 24 h, followed by simulation of I/R condition. Cells viability was then quantified by adding MTT solution (0.5 mg/ml in medium) for 3 h. experiments performed in triplicate(*n* = 3). Finally, formazan crystals were solubilized in 100 μl dimethyl sulfoxide (DMSO) at 37 °C for 10 min and absorbance was detected at 570 nm with an ELISA-plate reader (Model 550, Bio-Rad Laboratories, Inc).

### Simulation of H9C2 I/R model

For I/R model, H9C2 cells were subjected with the oxygen and glucose deprivation. First, cells were seeded into 96-well plates at the density of 10^4^ cells/well and incubated for 24 h. Then, standard medium (high glucose DMEM, 4.5 g/l) was replaced with the glucose-free DMEM, and cells were maintained in the oxygen-free incubator (95% N_2_, 5% CO_2_) at 37 °C for 2 h. Subsequently, medium was replaced with standard medium and cells were incubated in normoxic conditions (95% O_2_, 5% CO_2_) at 37 °C for another 24 h [19].

### Treatments

H9C2 cells were seeded in 6 well plates (10^4^ cells/well) and were divided into 4 groups as following:1) Control group, 2) Cells treated with PEF, 3) Cells cultured under I/R condition, 4) Cells pre-treated with PEF followed by I/R condition. To investigate the potentila reptans fraction effects, cell cultures in groups 2 and 4 were treated with PEF (200 µg/ml) 24 h before oxygen–glucose deprivation.

### RNA extraction and quantitative real-time PCR (qRT-PCR)

Total RNA was isolated from cells using Trizol reagent according to the manufacturer's instructions. Then, 1 μg of RNA samples was subjected to reverse transcription (RT)-PCR using cDNA Synthesis Kit (Thermo Fisher Scientific, Inc.). Real-time PCR was performed on each cDNA sample and quantified on a ABI 7300 system using SYBR Green PCR Master Mix (addbio, Korea). GAPDH was used as an internal reference gene. The primer sequences used for qPCR were as follows:

- H19 Forward:5′-ATCGGTGCCTCAGCGTTCGG-3′;
- Reverse:5′ CTGTCCTCGCCGTCACACCG-3′.
- MIAT Forward:5’-GGTTGGCTCTTTTGTCTTCCAGG-3′;
- Reverse:5′-GGCTCAGAGAAGTTGCTTGGTC-3′.
- GAPDH Forward:5′-AGCCACATCGCTCAGACAC-3′;
- Reverse:5′-GCCCAATACGACCAAATCC-3′. All experiments were performed in triplicate.

### Quantitative analysis of apoptosis

H9C2 cells were stained using Annexin V-FITC/PI detection kit (Biolegend, USA) according to the manufacturer’s protocol. Briefly, cells were harvested and washed with PBS and suspended in binding buffer containing 5 µl Annexin V-FITC and 10 μl propidium iodide (PI). Then, cells were incubated at room temperature in the dark for 15 min, followed by addition of 400 µl binding buffer. Finally, cells were analyzed by flow cytometry (BD Biosciences, Franklin Lakes, NJ).

### Molecular docking studies

#### Preparation of target proteins

A molecular docking analysis was carried out to estimate how the compounds of *Potentilla reptans* root interact with the target proteins (Caspase-8, NRF2, TNF-α, NF-κB, and SOD) [20]. The crystal structure of Caspase-8 receptor (PDB ID: 1QTN), NRF2 receptor (PDB ID:4L7B), TNF-α receptor (PDB ID: 2AZ5), NF-κB (PDB ID:1NFK) and Superoxide Dismutase I receptor (SOD) (PDB ID: 5YTO) were retrieved from Protein Data Bank. To prepare structure of the proteins follow steps as merging non-polar hydrogen, applying kollman charge using Auto Dock Tools (ADT). 100 genetic algorithm (GA) runs,27,000 maximum generations; a gene mutation rate of 0.02; and a crossover rate of 0.8 were set for Lamarckian genetic algorithm (LGA) searching method [20–22].

To evaluate the amino acid residues evolves in binding pocket of protein with compounds, we utilized Maestro 11.0 Schrodinger program to analyze how amino acid residues interact in the binding pocket of proteins with the mentioned natural compounds.

#### Ligand preparation

**Fig. 1:**
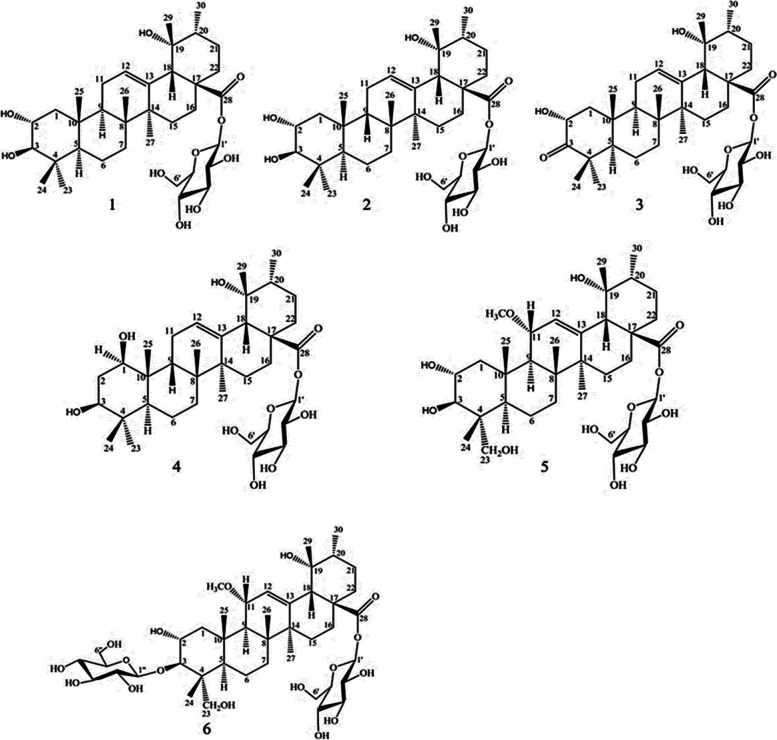
Structures of isolated compounds from *P. reptans* root

The 3-D structures of *P. reptans* root identified compounds (Fig. 1) [23] were taken from PubChem database. In the next step, energy minimization and optimization of each phytochemical were performed using Chem office suit.

### Statistical analysis

Data were analyzed by Graph Pad Prism 8 software (San Diego, CA). The differences between two groups were calculated by t-test and the significance of intergroup differences was evaluated by one-way analyses of variance (ANOVA). *P* values < 0.05 were the considered significant level. All experiments were conducted in triplicate, and data were expressed as mean ± SD.

## Results

### The effect of PEF on cell viability

**Fig. 2:**
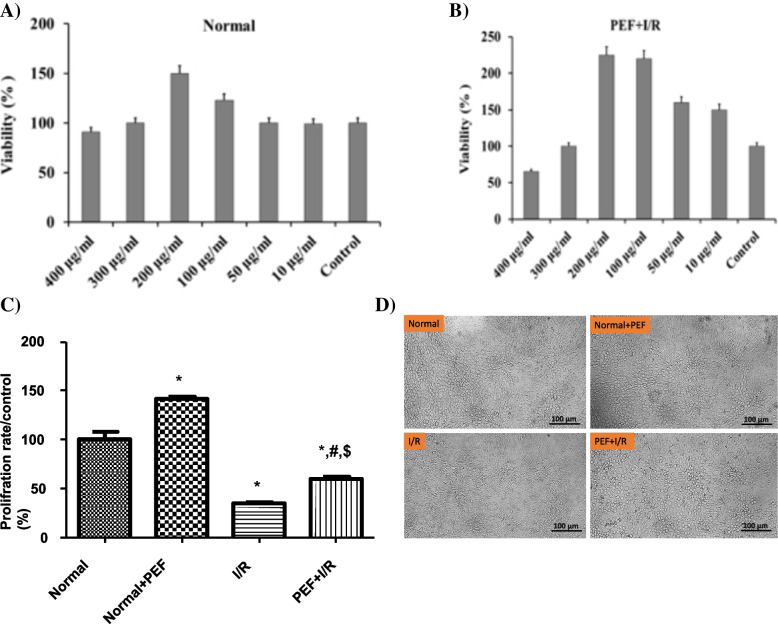
Effect of PEF on the viability of H9C2 cells under I/R conditions. **A** The H9C2 cells were treated with PEF from 10 to 400 µg/ml under normal. **B** The H9C2 cells were treated with PEF from 10 to 400 µg/ml under I/R conditions. **C** H9C2 cells were treated by PEF (200 µg/ml). **D** Light microscope image of H9C2 cells were treated by PEF (200 µg/ml). The experimental data are from three independent experiments (*n* = 3) and are presented as the mean ± SD. ^*^P < 0.05 vs. Normal, ^$^*P* < 0.05 vs. I/R, ^#^*P* < 0.05 vs. Normal + PEF

The cell viability of different concentrations of PEF on H9C2 cells was evaluated by MTT assay (Fig. 2A). H9C2 cell viability was significantly enhanced by the PEF under I/R conditions in a concentration-dependent manner up to 200 µg/ml. PEF at concentration of 200 µg/ml had the highest protective effects on I/R induced injury as EC_50_ (Fig. 2B, C and D). Therefore, this concentration was chosen for further experiments.

### Real time PCR analysis for lncRNAs

**Fig. 3:**
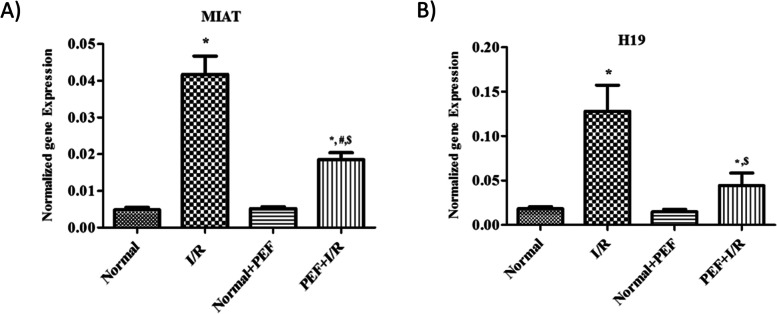
The expression levels of **A** H19 and **B** MIAT in H9C2 cells under I/R condition. Cells were pre-treated with PEF for 24 h, followed by simulation I/R. The experimental data are from three independent experiments (*n* = 3) and are presented as the mean ± SD. ^*^P < 0.05 vs. normal, ^$^*P* < 0.05 vs. I/R, ^#^*P* < 0.05 vs. Normal + PEF

Under I/R condition, PEF significantly decreased the expression of both lncRNAs H19 and MIAT vs. I/R group. Meanwhile, the expression of H19 and MIAT were increased in the PEF + I/R group as regards to the normal PEF group (Fig. 3A and B).

### Effects of PEF on cell apoptosis

**Fig. 4:**
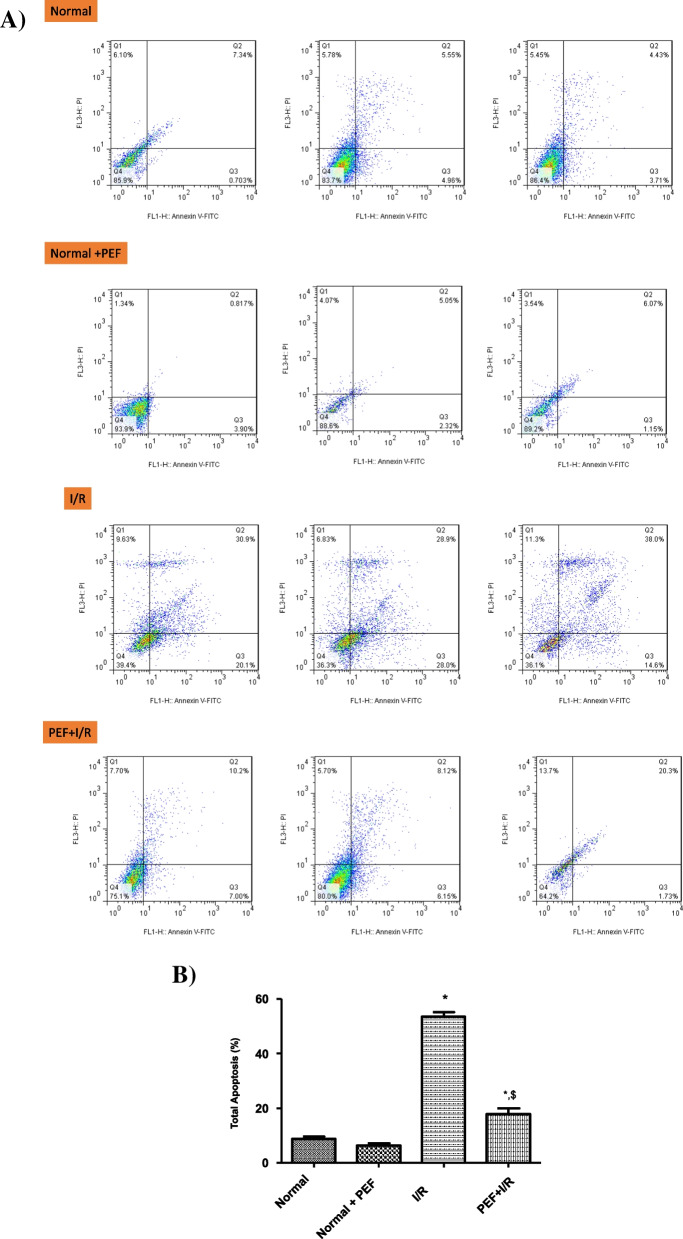
Effect of PEF on I/R-induced apoptosis in H9C2 cells. **A** Density plots of Annexin V-FITC/PI staining are based on flow cytometry analysis of no-treatment control and cells treated with PEF under I/R and normal condition. **B** Representing the apoptosis rates. Apoptosis decreased significantly when cells were treated with plant extract under I/R condition. The experimental data are from three independent experiments (*n* = 3). ^*^P < 0.05 vs. normal, ^$^*P* < 0.05 vs. I/R

In the present study, PEF reduced necrosis (5.78% before and 2.98% after the PEF treatment) and apoptosis in normal condition by 8.89% to 6.17%. Moreover, treatment with PEF before I/R significantly reduced (17.8 ± 3.94%) Annexin V/PI-positive H9C2 cells compared with I/R (53.5 ± 3.05%) group, as shown in (Fig. 4A & B).

### Molecular docking

According to Table 1, the results revealed that the triterpenoids of *P. reptans* have good potential for Caspase-8, NRF2, SOD, TNF-α and NF-κB receptors. They are also more specific and effective due to their low binding energy at Caspase-8 and NRF2 receptors.

**Table 1:** Molecular docking simulations results for the compounds and Caspase-8, NRF2, SOD, TNF-α and NF-κB receptors

Compounds	PDB ID: 1QTN	PDB ID: 4L7B	PDB ID: 5YTO	PDB ID: 2AZ5	PDB ID: 1NFK
	**Caspase-8**	**NRF2**	**SOD**	**TNF-α**	NF-κB
**1**	-7.9	-8.2	-8.1	-7.6	-7.3
**2**	-8.1	-8.1	-8.5	-7.8	-7.2
**3**	-8.3	-9.4	-9.2	-7.7	-7.8
**4**	-8.6	-8.7	-8.8	-8.1	-7.3
**5**	-8.3	-8.9	-8.3	-8.4	-7.6
**6**	-9.3	-8.4	-9.6	-8.0	-7.5

**Fig. 5:**
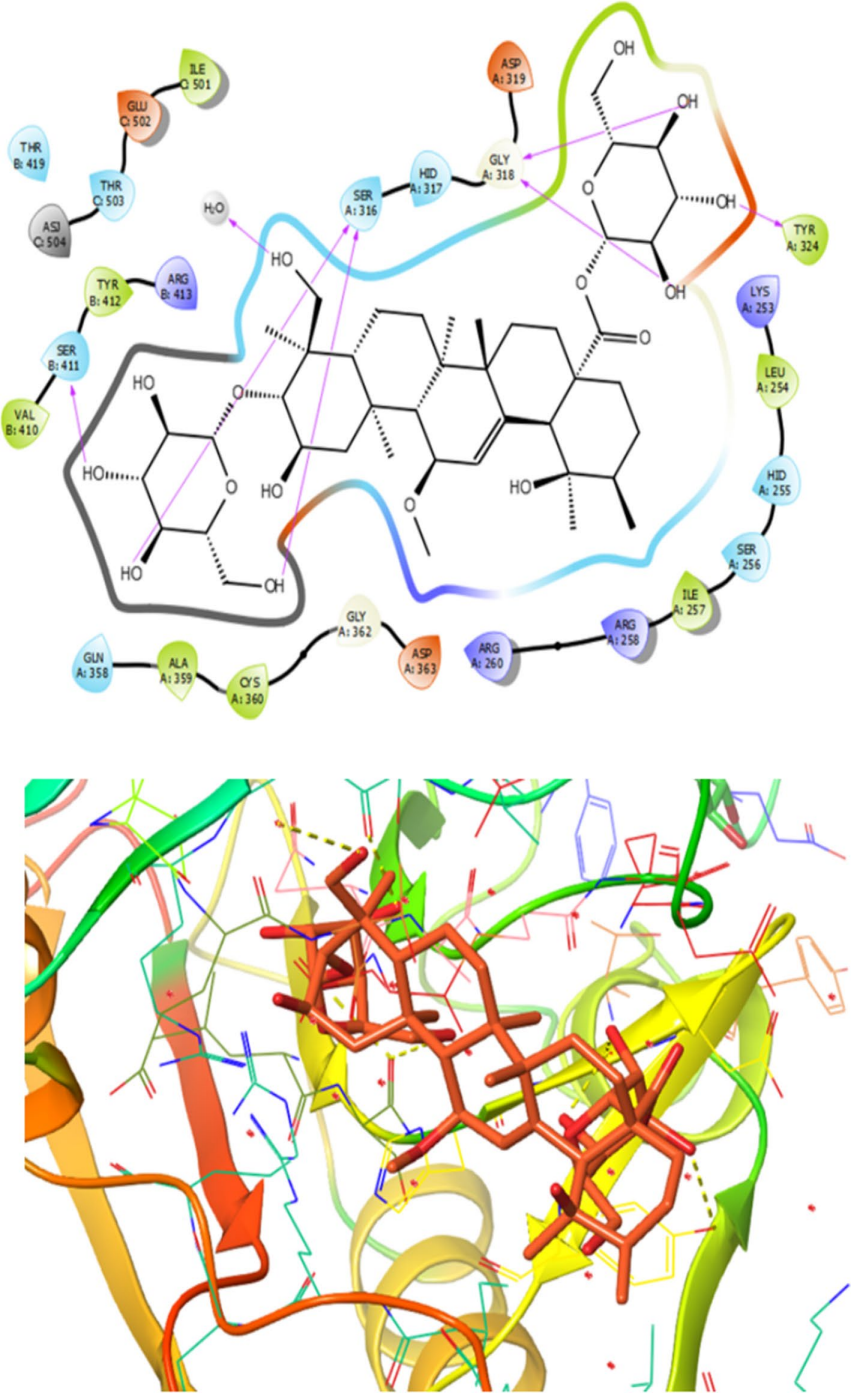
Presentation of 2D and 3D models of interactions between compound **6** and Caspase-8 receptor (PDB ID: 1QTN)

Furthermore, binding energy for the compound **6** in the active site of proteins (1QTN, 4L7B, 2AZ5, 1NFK and 5YTO) were -9.3, -8.4, -8.0, -7.5 and -9.6 (kcal/mol), respectively. Figure 5 depicted the 2D and 3D model of interactions between compound **6** and target of 1QTN indicating high binding affinity toward active site of the target.

As depicted in Fig. 5 the analysis of the interactions between compound **6 **and target of structure of Caspase-8 receptor (PDB ID: 1QTN illustrated that amino acid residues such as Val410, Tyr412, Ile501, Tyr324, Leu254, Ile257, Ala359 and Cys360 formed a hydrophobic pocket interacting with compound **6** by hydrophobic contacts Additionally, compound **6** indicated polar interactions with amino acid residues like Gln358, Ser411, Thr503, Thr419, Glu502, Arg413, Ser316, Hid317, Asp363, Arg260, Asp319, Lys253, Hid255, Ser256, Arg258. Moreover, it was found that amino acid residues of Gln358, Cys360, Ser316 and Ser411 had interaction with hydroxyl group of compound **6** at distances 3.0, 2.7, 3.1, 2.8, 2.2, 2.1, and 1.6 Å, respectively (Fig. 5). Therefore, hydroxyl group has important role in increasing interaction with binding pocket of target.

**Fig. 6:**
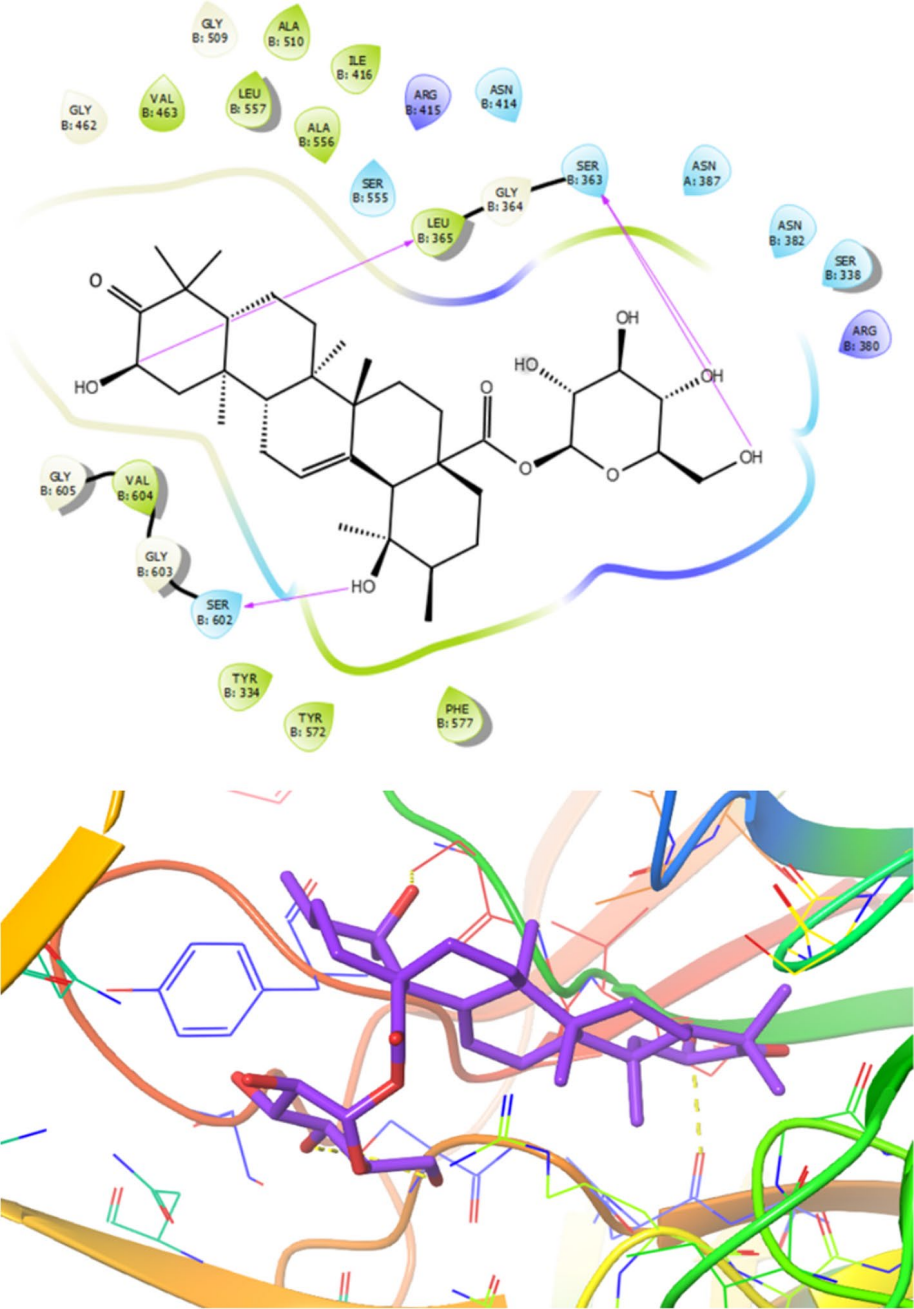
Presentation of 2D and 3D models of interactions between compound **3** and Nrf2 receptor (PDB ID:4L7B)

As illustrated in Fig. 6, on the basis of obtained docking results, compound 3 revealed the lowest dock score (binding energy) and best orientations toward NRF2 receptor (PDB ID:4L7B). Amino acid residues such Val463, Leu557, Ala510, Ile416, Ala556, Leu365, Phe577, Tyr572, Tyr334, Gly603, Gly605 and Val604 involved in the active site of the target through hydrophobic interaction and also compound **3** formed polar interaction with residues like Ser602, Ser555, Arg415, Asn414, Ser363, Asn387, Asn382, Ser338 and Arg380. Furthermore, functional groups of the compound **3** formed six hydrogen bond with residues Leu365, Val604, Arg415Ser363 and Ser602 at distances 2.44, 1.932.21, 2.19, 1.65 and 2.14 Å, respectively (Fig. 6).

**Fig. 7:**
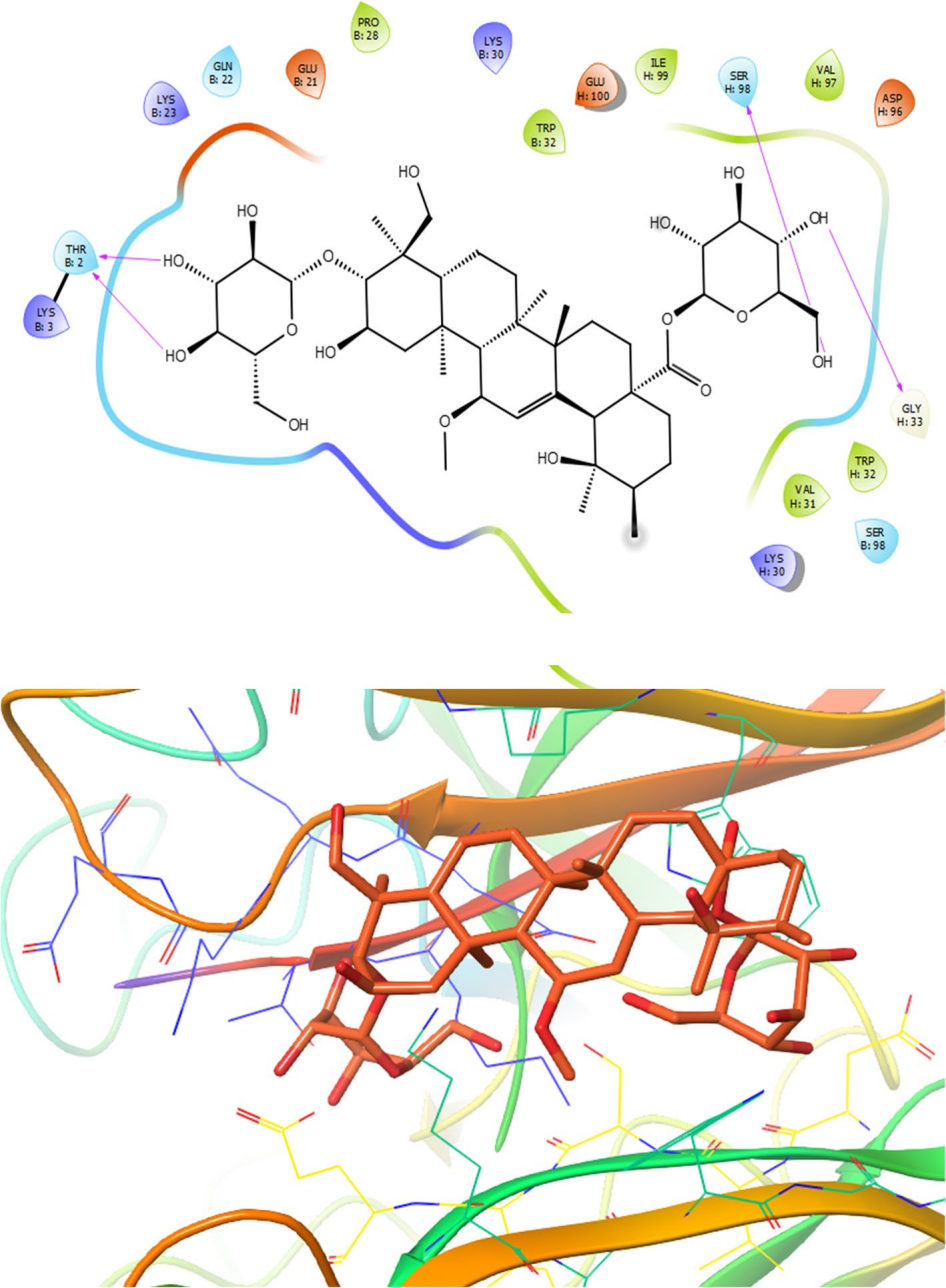
Presentation of 2D and 3D models of interactions between compound **6** and SOD receptor (PDB ID: 5YTO)

As illustrated in Fig. 7, on the basis of obtained docking results, compound 6 revealed the lowest dock score (binding energy) and best orientations toward SOD receptor (PDB ID: 5YTO). Amino acid residues such as Pro28, Trp32, Ile99, Val97 and Val31 involved in the active site of the target through hydrophobic interaction and also compound **6** formed polar interaction with residues like Thr2, Gln22, Lys3, Lys23, Lys30, Glu21, Glu100, Ser98, Asp96 and Lys30. Furthermore, functional groups of the compound **6** formed six hydrogen bond with residues Thr2, Ser98, Val97and Gly33 at the distances 2.15, 2.39, 1.78, 1.59, 3.09 and 2.08 Å, respectively (Fig. 7).

**Fig. 8:**
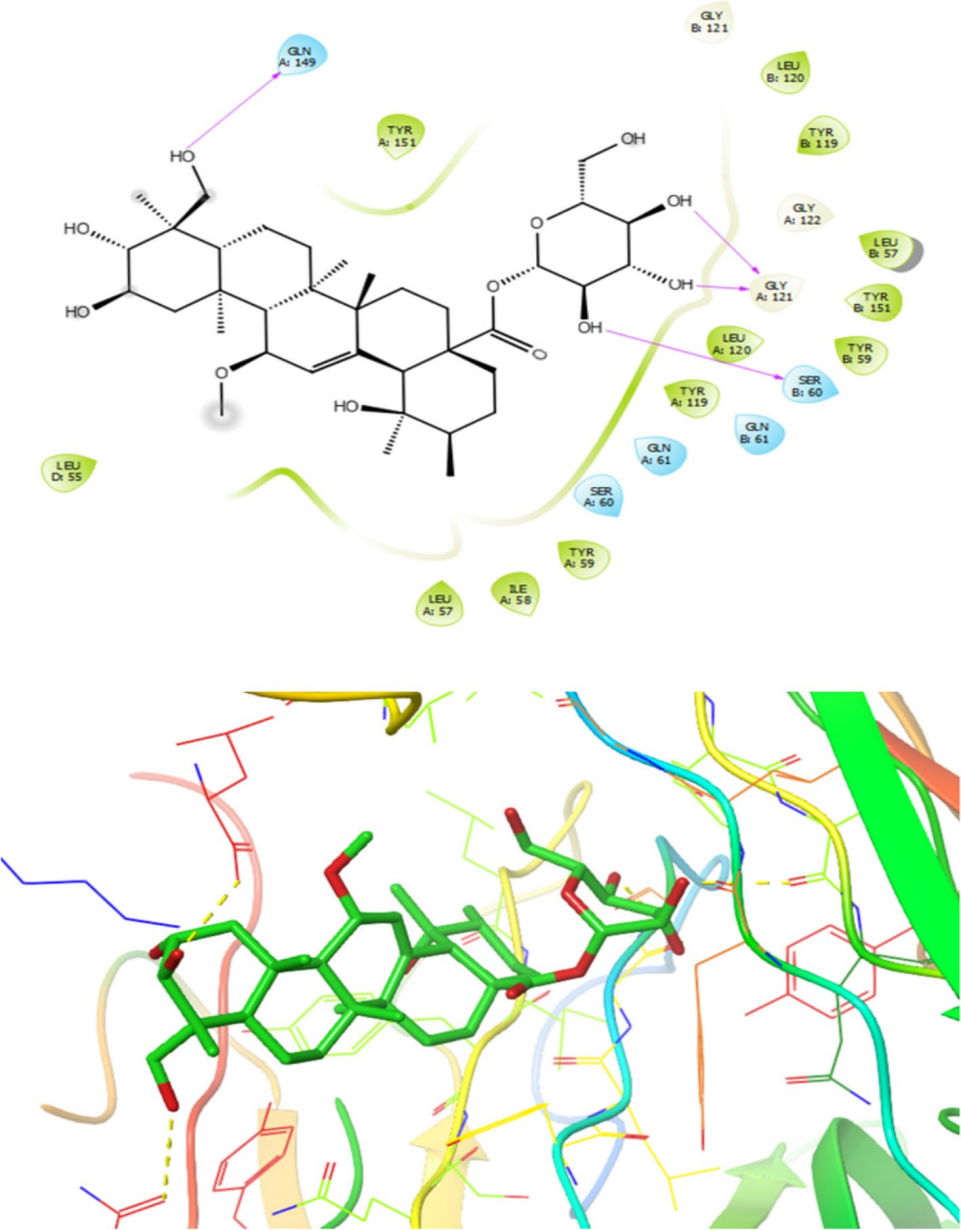
Presentation of 2D and 3D models of interactions between compound **5** and TNF-α receptor (PDB ID:2AZ5)

**Fig. 9:**
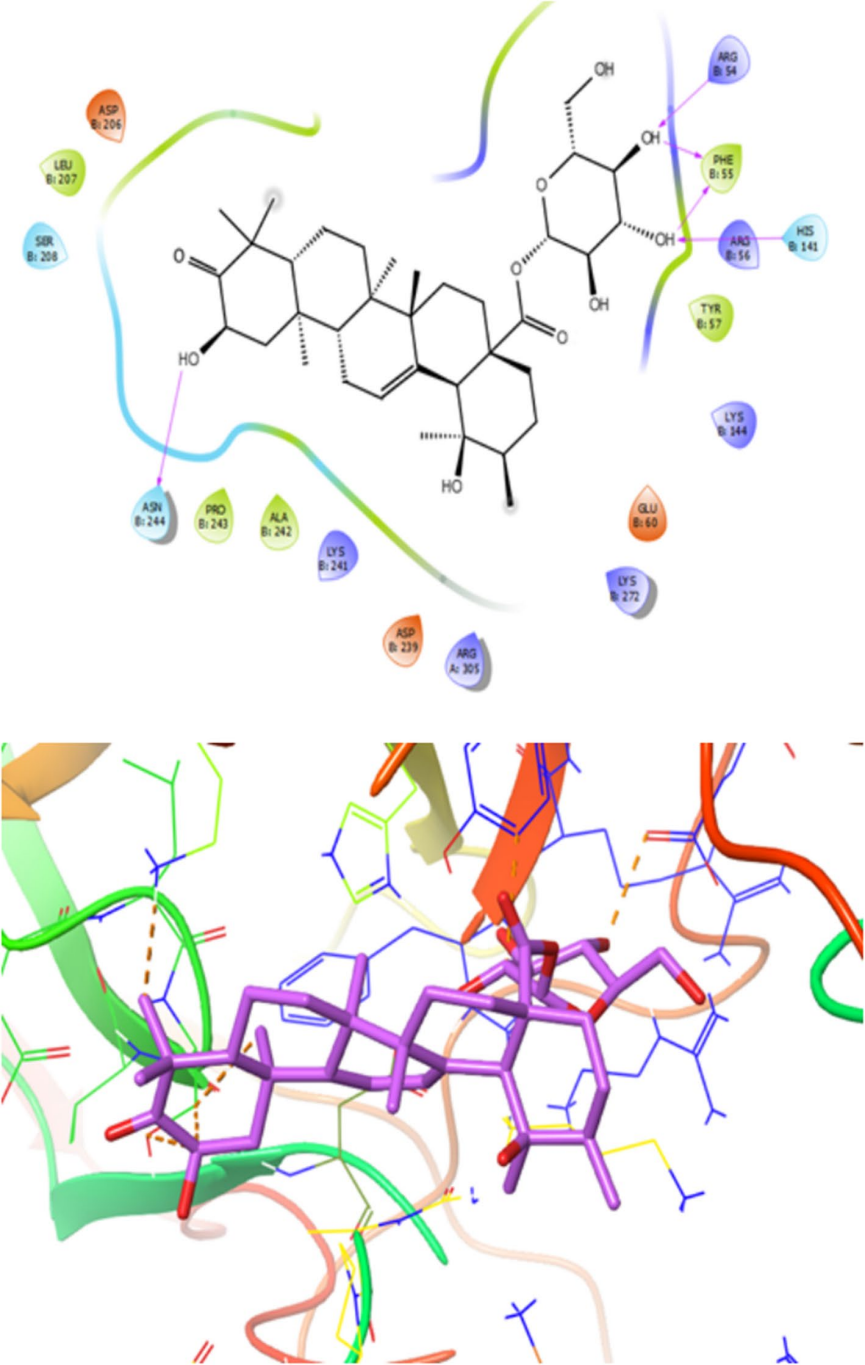
Presentation of 2D and 3D models of interactions between compound **3** and NF-κB receptor (PDB ID:1NFK)

The anti-inflammatory effects of triterpenoid compounds are mediated by inhibition of TNF-α and NF-κB (Figs. 8 and 9). The analysis of the interactions between compound **5** and target of TNF-α receptor (PDB ID: 2AZ5) illustrated that amino acid residues such as leu120, Tyr119, Tyr59, Leu57 and Ile58 in chain A and also Leu120, Tyr119, Leu57 and Tyr59 in chain B involved in the active site of the target through hydrophobic interaction and also the compound **5** formed polar interaction with residues like Gln149, Gly121, Ser60 and Gln61 in chain A and also Gln61 and Ser60, in chain B furthermore, functional group of the compound5 formed six hydrogen bond with residues Gln149, Gly121, Leu157, Tyr151 and Ser60 at the distances of 1.16, 1.80, 2.36, 1.97,2.62 and 1.72 Å, respectively (Fig. 8). Therefore, hydroxyl group has important role in increasing interaction with binding pocket of the target.

As illustrated in Fig. 9, on the basis of obtained docking results, compound **3** revealed the lowest dock score (binding energy) and best orientations toward NF-κB receptor (PDB ID:1NFK). Amino acid residues such as Leu207, Pro243, Ala242, Tyr57, Phe55involved in the active site of the target through hydrophobic interaction and also compound3 formed polar interaction with residues like Lys241, Arg305, Lys272, Asp239, Glu60, Lys144, Arg56 His141, Ser208 and Asp206. Furthermore, functional groups of the compound **3** formed six hydrogen bond with residues Asn244, Arg54, Phe55 and His141at distances 2.46, 2.77, 2.65, 2.01, 1.57and 2.44 Å, respectively (Fig. 9).

## Discussion

In the current study, PEF exerted a preconditioning effects and enhanced cell viability of H9C2 cells in both normal and I/R conditions. The benificial preconditioning effects of PEF with EC_50_ index (200 μg/ml) in the MTT assay showed an intense proliftraion effect of H9C2 cells compared with I/R and normal conditions. Our data exhabited the anti-hypoxic effcet of preteratment of PEF, mediated via reducing MIAT and H19 genes, which finally led to the anti-apoptotic effects. In addition, molcular dokcing analysis showed that isolated triterpenoid compounds of PEF, especially compound **3** and **6**, have aroused much interest for inhibiting the active site of caspase-8, TNF-α and NF-κB as well as activating NRF2 and SOD targets.

Some other studies reported that *P. reptans* extract exhibit cardioprotective and anti-apoptotic effects in myocardial cell lines under hypoxic and ischemic conditions [7, 8, 19, 24, 25]. In vitro and in vivo studying showed that the extract of *Potentilla* genus significantly enhanced the survival of heart cells against hypoxia-induced cardiomyocyte injury via reducing the level of LDH, and CK, preventing MDA production, increasing SOD activity and anti-apoptotic effects in cardiomyocyte [14]. The study showed the anti-apoptotic and cardioprotective effects of *P. reptans* extract against myocardial ischemia/reperfusion injury via inhibiting the expression of Caspase-9 and Caspase-3 [18]. Recent updates, revealed the ischemic preconditioning and cardioprotective effects of total extract and ethyl acetate fraction from *P. reptans* root via NO release, an activation of NRF2 and antioxidant pathways, a reduction in apototic indexs, boosting cardiac function and anti-arryhthmic properties in an isolated rat heart ischemia/reperfusion model [2, 4].

This study revealed the regulatory effects of PEF on lncRNA H19 and MIAT in I/R-induced H9C2 injury. A growing body of evidence shows that H19 and MIAT mediated pathological processes of myocardial injuries against I/R and hypoxia [12, 13]. Under hypoxia, the overexpression of H19 lead to a reduction in hypoxia‑induced apoptosis in H9C2 cells via activation of the PI3K/AKT and the ERK/p38 pathways [11]. In addition, lncRNA H19 may exert its cardioprotective and anti-apoptotic effects through a suppretion of miR-22-3p, as a pivotal miRNAs, in ischemic myocardial injuries [8]. By contrast, the knockdown of H19 increased apoptosis in the H9C2 cardiomyoblast cell line [11]. Therefore, H19 plays a critical role in diminishing cardiac injuries during I/R. In this study, the expression of H19 was increased in the both (PEF + I/R) and I/R groups against normal condition. Interestingly, decreased expression of H19 was observed in the (PEF + I/R) group compared with I/R, suggesting that PEF may provid a cardioprotection by its preconditioning effects and inducing H19 as balanced level for preserving against I/R injury. In addition, it has been reported that the aberrant exppression of MIAT in cardiac tissue triggerd incidence of apoptosis and necrosis during I/R [8, 12–14]. Morever, silencing lncRNA MIAT increased H9C2 cell viability and heart function as well as reversed H/R-induced apoptosis in cardiomyocyte [26]. The result of this study revealed that the expression of MIAT was significantly increased in I/R condition against normal group, while the administration of PEF was not changed the MIAT level of normal group which in line with the other studies [25, 26]. Meanwhile, PEF preconditioning strongly inhibited the expression of MIAT that burstly induced by I/R in the H9C2 cells.

Furthermore, the results showed that PEF preconditioning improved H9C2 cell viability in normal condition via decrasing the cell dispersion in both necrosis and late apoptotic phases. In addition, PEF preconditioning (PEF + I/R) remarkably reversed adverse effects of I/R and induced beneficial features on H9C2 cells by reducing the early and late phases of apoptosis (Fig. 4A).

In the process of cardiovascular diseases, the oxidative stress, I/R and hypoxia initiate apoptosis and necrosis in cardiomyocyte [2]. Consistent with this, ethyl acetate fraction of *Potentilla reptans* root revealed anti-apoptotic effects via increasing Bcl-2, and reducing Bax and caspase3 level in the both pre/postconditioning rat heart I/R model [2, 4]. In line with our result, the previous study indicated that MIAT knockdown reduced cerebral I/R injury and apoptosis in rat cortical neurons through free radical scavenging and diminishing caspase-3 and Bax as apoptotic markers as well as increasing anti-apoptotic Bcl-2 protein [24]. However, it would be plausible that PEF could suppress apoptosis in the cell cycle progression through the reduction of MIAT expression and H19 induction as well as involving their target axis.

In the next step, we determined that isolated compounds of PEF activated the NRF2 pathway and SOD by molecular docking study. The mechanism of NRF2 is one of the major cellular pathways in the combating against oxidative stress and regulation of cellular redox. These compounds are able to increase the expression of the transcription factor NRF2 and subsequently the induction of antioxidant enzymes in heart cells [23, 27]. Also, induction of superoxide dismutase (SOD) protects heart cells from damage by neutralizing free radicals. Decreased plasma activity of this enzyme increases the lipid peroxidation of endothelial cell membranes and provides the basis for cardiovascular problems. A comparison of molecular docking results revealed that compound **6** and **3** had lower binding energy to the targets structure while showing a strong interaction towards the binding pocket of the targets and inhibit more effectively the active site of the protein than the other compounds. Therefore, the triterpenoids of PEF activate intracellular pathways by increasing NRF2 expression and regulate ROS mechanisms.

It has been proposed that PEF exerts its anti-apoptotic effects due to active compounds such as triterpenoids which inhibit Caspase-8 for protecting heart cells from I/R injuries. The comparison of chemical structure of compounds exhibited that hydroxy group of compounds formed good interactions with amino acid residues of receptors and occupy active site of targets. Overall, the hydrogen, hydrophobic and polar interactions might act as an important role in occupation of the binding pocket. [28, 29]. Likewise, molecular orientation can be effective in binding affinity. Accordingly, compound **3** and **6** could be a potential inhibitor of the Caspase-8, TNF-α and NF-κB at the binding site in line with the measured genes.

Additionally, pharmacokinetic and drug likeliness parameters of the compounds including absorption, distribution, metabolism, excretion and toxicity (ADMET) were previously reported [30].

Taken together, the present study indicated that PEF preconditioning has anti-apoptotic and protective effects on H9C2 cell viability in I/R condition. Moreover, it can reduce the expression of lncRNA MIAT and apoptosis in I/R model. Additionally, PEF preconditioning exerts cardioprotective effects via regulating H19 expression. Thus, PEF preconditioning can be suggested asa potential candidate for the regulation of lncRNAs during I/R and hypoxia injuries. However, it is necessary further studies to indicate the cross talk between genes involved in I/R injury and isolated ingredients of PEF.

## Conclusions

PEF preconditioning reveals the cardioprotective and anti-apoptotic properties in the H9C2 through amplification of the NRF2 pathway and SOD, regulation of H19 expression, and suppress of MIAT gene, resulting in decreased I/R injury, inflammation and oxidative stress. Our findings postulate that PEF can be as a potential preconditioning agent as well as lncRNAs H19 and MIAT regulator in clinical practice after passing preclinical and clinical studies The study of molecular docking exhibits compound 3 and 6 containing hydroxy groups has a strong binding affinity with NRF2, SOD, Caspase-8, TNF-α, and NF-κB receptors in comparison with the other compounds. As a result, compound **3** and **6** could be a potential activator of the NRF2 and SOD receptors and also potential inhibitor of the apoptosis (Caspase-8) and inflammatory (TNF-α and NF-κB) receptors at the binding site.

## Data Availability

The datasets used and/or analysed during the current study are available from the corresponding author on reasonable request.
